# Spider mites suppress tomato defenses downstream of jasmonate and salicylate independently of hormonal crosstalk

**DOI:** 10.1111/nph.13075

**Published:** 2014-10-08

**Authors:** Juan M Alba, Bernardus C J Schimmel, Joris J Glas, Livia M S Ataide, Maria L Pappas, Carlos A Villarroel, Robert C Schuurink, Maurice W Sabelis, Merijn R Kant

**Affiliations:** 1 Department of Population Biology, Institute for Biodiversity and Ecosystem Dynamics, University of AmsterdamPO Box 94240, 1090 GE, Amsterdam, the Netherlands; 2 Department of Entomology, University of ViçosaViçosa, Brazil; 3 Department of Agricultural Development, Laboratory of Agricultural Entomology and Zoology, Democritus University of ThracePantazidou 193, 68 200, Orestiada, Greece; 4 Department of Plant Physiology, Swammerdam Institute for Life Sciences, University of AmsterdamPO box 94215, 1090 GE, Amsterdam, the Netherlands

**Keywords:** defense suppression, herbivore communities, hormonal crosstalk, jasmonic acid (JA), salicylic acid (SA), *Solanum lycopersicum* (tomato), *Tetranychus* spp. (spider mite)

## Abstract

- Plants respond to herbivory by mounting a defense. Some plant-eating spider mites (*Tetranychus* spp.) have adapted to plant defenses to maintain a high reproductive performance. From natural populations we selected three spider mite strains from two species, *Tetranychus urticae* and *Tetranychus evansi*, that can suppress plant defenses, using a fourth defense-inducing strain as a benchmark, to assess to which extent these strains suppress defenses differently.
- We characterized timing and magnitude of phytohormone accumulation and defense-gene expression, and determined if mites that cannot suppress defenses benefit from sharing a leaf with suppressors.
- The nonsuppressor strain induced a mixture of jasmonate- (JA) and salicylate (SA)-dependent defenses. Induced defense genes separated into three groups: ‘early’ (expression peak at 1 d postinfestation (dpi)); ‘intermediate’ (4 dpi); and ‘late’, whose expression increased until the leaf died. The *T. evansi* strains suppressed genes from all three groups, but the *T. urticae* strain only suppressed the late ones. Suppression occurred downstream of JA and SA accumulation, independently of the JA–SA antagonism, and was powerful enough to boost the reproductive performance of nonsuppressors up to 45%.
- Our results show that suppressing defenses not only brings benefits but, within herbivore communities, can also generate a considerable ecological cost when promoting the population growth of a competitor.

## Introduction

Higher plants possess sophisticated means to prevent or hamper herbivore feeding (Walling, 2000; Wu & Baldwin, 2010). Such defenses can be constitutive and/or induced upon attack by herbivores. In general, induced defenses may include morphological reinforcements as well as the accumulation of toxins and inhibitors of herbivore digestion (Kessler & Baldwin, 2002), but may also involve hypersensitive responses (Klingler *et al*., 2009) and resource allocation (Gomez *et al*., 2012). The first critical step to mount antiherbivore defenses is the perception of herbivory, but how this takes place and whether receptors are involved is not well understood (Bonaventure *et al*., 2011). It is clear that some characteristics of the response can be attributed to mechanical feeding damage (Mithöfer *et al*., 2005) but others can only be attributed to herbivory-derived signals referred to as elicitors (Howe & Jander, 2008). Most of these emanate from herbivore saliva or regurgitant and, when applied as pure compounds, elicit defined herbivory-induced changes, such as phytohormone accumulation, transcription of defense genes, and emission of volatiles (Wu & Baldwin, 2010).

The central regulators of plant defense responses are a set of phytohormones that mediate between signal recognition and activation of defenses. Although most of the known plant hormones have been found to influence the establishment of defenses in one way or another (Pieterse *et al*., 2012), there are three, jasmonic acid (JA), salicylic acid (SA) and ethylene (Et), which play primary roles, as interference with their biosynthesis or perception results in strong defense deficiencies (Wu & Baldwin, 2010). While JA, SA and Et have distinct effects on the type of defenses a plant displays, they also modulate each other's individual actions, that is, ‘crosstalk’ (Pieterse *et al*., 2009), in a nonlinear way (Mur *et al*., 2006). While SA is essential for defense against biotrophic pathogens (Vlot *et al*., 2009), JA and, in particular, its amino acid conjugate JA-isoleucine (JA-Ile) are essential for defenses against herbivores (Howe & Jander, 2008) and necrotrophic pathogens (Glazebrook, 2005), whereas Et most probably modulates these two (Diezel *et al*., 2009). Defense responses induced by stylet-feeding herbivores appear to involve a cocktail of JA and SA responses (Kaloshian & Walling, 2005). In tomato (*Solanum lycopersicum*) JA accumulation is upstream of the expression of several defense genes, commonly used as markers for JA defenses, such as *Wound-Induced Proteinase Inhibitor I* (*WIPI-I*) and *II* (*WIPI-II*) (Farmer *et al*., 1992), *Threonine Deaminase 2* (*TD-2*) (Gonzales-Vigil *et al*., 2011) and the activities of defensive enzymes such as polyphenol oxidases (PPOs) and peroxidases (Felton *et al*., 1989). SA defenses, in turn, are marked by the expression of pathogenesis-related (PR) proteins (Van Loon & Van Strien, 1999) and, in many different plant species, by the accumulation of reactive oxygen species (ROS), sometimes followed by apoptosis (Walling, 2000). Collectively, these are referred to as direct defenses. In addition, JA regulates the biosynthesis and release of an induced blend of volatiles, in part depending on SA (Ament *et al*., 2004, 2010), which can attract foraging natural enemies of herbivores and is therefore referred to as indirect plant defense (Kant *et al*., 2009).

The guild of stylet-feeding arthropods can be divided into two subguilds, those that feed predominantly on vascular sap, usually phloem sap, and those that feed from cytoplasm only (Miles, 1972). The latter applies to spider mites (*Tetranychus* ssp.): the adults use stylets of *c*. 150 μm long for lacerate-and-flush feeding on mesophyll cells, predominantly parenchyma, of which they can empty up to 18–22 min^−1^ (Jeppson *et al*., 1975), leading to *c*. 1 mm^2^ of visible chlorotic leaf surface area per adult mite d^–1^ on tomato (Kant *et al*., 2004). The two-spotted spider mite *T. urticae* is highly polyphagous and has been recorded to feed from over 1100 plant species, among them tomato (Dermauw *et al*., 2012). This mite species is endemic to Europe. The red spider mite, *Tetranychus evansi*, is a specialist on Solanaceae in Brazil and Africa and a recent invasive pest in Europe (Boubou *et al*., 2012), where it has extended its host range and has displaced *T. urticae* on several host plant species in southern Europe (Ferragut *et al*., 2013). Adult females of both species produce, on tomato, between five and 15 eggs d^−1^ which will develop into fertile adults within *c*. 2 wk, resulting in exponential population growth and, subsequently, host–plant overexploitation (Sarmento *et al*., 2011a). Spider mites produce silk webbing across the host-plant surface which shields them and their eggs from natural enemies. However, while biological control of *T. urticae* is well feasible, that of *T. evansi* is troublesome, as the webbing it produces is extraordinarily dense while, in addition, many biological control agents have a poor reproductive performance when preying on it (Sarmento *et al*., 2011b; Navajas *et al*., 2013).

When feeding on tomato leaves, most genotypes of *T. urticae* simultaneously induce expression of the JA- and SA-dependent marker genes *WIPI-II* and *PR-P6*, respectively (Li *et al*., 2002; Ament *et al*., 2004; Kant *et al*., 2004). However, some genotypes of *T. urticae* and *T. evansi* were found to suppress expression of these marker genes (Kant *et al*., 2008; Sarmento *et al*., 2011a). The use of the JA-perception mutant *jasmonic acid-insensitive-1* (*jai-1*; Li *et al*., 2004) and of the biosynthesis mutant *defenseless-1 (def-1*; Li *et al*., 2002; Ament *et al*., 2004; Kant *et al*., 2008) has demonstrated that spider mites reach their maximal reproductive performance in the absence of JA signaling, while on *35S::Prosystemin* tomato, which is primed to display exceptionally strong JA defenses (Chen *et al*., 2006; Kandoth *et al*., 2007), reproductive performance is minimal. Although this strongly suggests that JA defenses are key anti-mite defenses for tomato, it appears that some spider mites have acquired resistance to them (Kant *et al*., 2008). However, such direct resistance against JA defenses was absent in the defense-suppressing *T. urticae* genotype (Kant *et al*., 2008). Taken together, these data suggest that the traits that enable some mite genotypes to suppress plant defenses are not likely to be allelic variants of the same traits that enable other mite genotypes to resist the same defenses.

Suppression of plant defenses is a phenomenon that is especially well known from plant pathogens (Abramovitch *et al*., 2006; Kamoun, 2006), but also herbivores, such as nematodes (Haegeman *et al*., 2012) and insects (Musser *et al*., 2002, 2005; Will *et al*., 2007; Zarate *et al*., 2007; Weech *et al*., 2008; Zhang *et al*., 2009, 2011; Bos *et al*., 2010; Consales *et al*., 2012; Stuart *et al*., 2012; Wu *et al*., 2012), were found to manipulate plant defenses. Spider mites and insects do not share a recent history: the Chelicerates (among which the mites evolved) and the Uniramians (among which the insects evolved) diverged early in the arthropod lineage, probably well over 400 million yr ago, from an aquatic ancestor (Weygoldt, 1998), suggesting that traits that allow some of the current plant-eating insect and mite species to suppress host defenses may have evolved independently. Hence we reasoned that the distinct intraspecific and heterospecific variation among Tetranychid mites (Matsushima *et al*., 2006; Kant *et al*., 2008; Sarmento *et al*., 2011a) forms an ideal basis for assessing some of the ecological costs and benefits of defense suppression within herbivore communities and for determining which processes are targeted by suppression. Therefore, we selected several putative defense-suppressing spider mites from natural populations, determined how tomato plants responded to them, and to what extent these responses modulate the mite's interactions with its natural defense-inducing competitors within two species communities.

## Materials and Methods

### Plants

Tomato (*Solanum lycopersicum* L. cv Castlemart, *35S::prosystemin* and *def-1*) and bean (*Phaseolus vulgaris* L. cv Speedy) were germinated and grown in a glasshouse (16 : 8 h, 25 : 18°C, day : night, 50–60% relative humidity (RH)). Experiments involving plants were carried out in a climate room (25°C, 16 : 8 h, light : dark, 60% RH, 300 μmol m^−2^ s^−1^), to which plants were transferred 3 d in advance.

### Spider mites

*Tetranychus evansi* Baker & Pritchard Viçosa-1 (Supporting Information, Notes S1; Fig. S1a; Sarmento *et al*., 2011a), *T. evansi* Algarrobo-1 (Fig. S1b; this paper), *T. urticae* Koch DeLier-1 (Fig. S1c; this paper, see the section ‘Selection of *T. urticae* DeLier-1’) and *T. urticae* Santpoort-2 (Fig. S1d; ‘KMB’ in Kant *et al*., 2008) were reared on detached leaves of *S. lycopersicum* cv Castlemart (for *T. evansi*) or *P. vulgaris* cv Speedy (for *T. urticae*) in a climate room (25°C 16 h : 8 h, light : dark, 60% RH, 300 μmol m^−2^ s^−1^). The species identity of all four strains was confirmed on the basis of a phylogenetic reconstruction using their mitochondrial cytochrome oxidase subunit 1 (COI) sequences (Fig. S2). For all infestation experiments and performance assays, we used adult female mites (3 ± 1 d old).

### Selection *of T. urticae* DeLier-1

Adult *T. urticae* females were collected from three natural populations in the Netherlands in 2009: 125 individuals from spindle tree (*Euonymus europaeus* L.), 64 from deadnettle (*Lamium album* L.) and 50 from castor oil (*Ricinus communis* L.) plants. Mites were individually transferred to *def-1* leaves. Their virgin female offspring (F_1_) were separated again and allowed to produce eggs on *def-1*. Mothers with a high reproductive performance (≥ 20 eggs per 4 d) were backcrossed with their sons for two more generations to F_3_ (hereafter referred to as ‘strains’). The fecundity of adult females of all strains was subsequently assessed on *def-1*, wild-type (WT) and *35S::Prosystemin* tomato plants to identify JA defense-suppressing mites (Kant *et al*., 2008). This yielded one putative suppressor strain from the spindle tree population; three from the deadnettle population and one from the castor oil population (Fig. S3a). After comparing the expression levels of *Proteinase Inhibitor IIf* (*PI-IIf*) induced by these strains on tomato with those induced by the benchmark inducer strain *T. urticae* Santpoort-2 and in uninfested controls, we selected the strain that gave the smallest increase in *PI-IIf* transcript abundances for further experiments; this was *T. urticae* DeLier-1 (Fig. S3b).

### Performance assays for individual spider mite strains

To establish whether our spider mite strains are affected by JA-mediated defense responses, we assessed their performance on WT and *def-1* tomato plants. Adult females were transferred to 21-d-old tomato plants (Methods S1): five mites per leaflet; three leaflets per plant; six plants per treatment. After 4 d, the number of eggs was recorded using a stereo microscope. This experiment was repeated three times. The total number of eggs per female were analyzed for each tomato genotype, and statistically analyzed using the Student's *t*-test (PASW Statistics 17.0; SPSS Inc., Chicago, IL, USA).

### Performance assay for two spider mite strains sharing a leaflet (coinfestation)

To assess the extent to which one strain can influence the reproductive performance of another strain, we followed the setup used in Kant *et al*. (2008). Leaflets of 21-d-old intact tomato plants were divided into two using a lanolin barrier. Five *T. urticae* Santpoort-2 females were transferred to the tip-half of the leaflet, whereas the petiolule-half was infested with 15 mites from one of the suppressor strains (five + 15 mites per leaflet; three leaflets per plant; six plants per treatment). After 4 d, the number of eggs laid by the five *T. urticae* Santpoort-2 females at the tip was recorded. This experiment was repeated three times. The average number of eggs per female 4 d^−1^ was analyzed using ANOVA and the means of each group were compared by least significant difference (LSD) *post hoc* test using PASW Statistics 17.0.

### Phytohormone and gene expression assay on leaflets infested with 15 mites (time course)

Leaflets of 21-d-old tomato plants were infested with adult female spider mites: 15 mites per leaflet; three leaflets per plant; 12 plants per treatment. At 1, 4 and 7 d postinfestation (dpi); four infested plants from each treatment and four control plants were sampled: infested leaflets and corresponding noninfested leaflets of control plants were excised (without petiolule), flash-frozen in liquid nitrogen and stored at −80°C until we extracted phytohormones and mRNA. The three leaflets obtained from the same plant were pooled to form one biological replicate. Under these standard experimental conditions (Kant *et al*., 2004), leaflets with *T. urticae* Santpoort-2 enter senescence 8–9 dpi and die *c*. 11–12 dpi (J. M. Alba & B. C. J. Schimmel pers. obs.).

### Phytohormone and gene expression assay on leaflets simultaneously infested with mites from two different strains (coinfestation)

Leaflets of 21-d-old tomato plants were infested with adult female spider mites: five to 30 mites per leaflet; three leaflets per plant; six to 10 plants per treatment. At 7 dpi, leaflets were harvested and stored as described earlier. The three leaflets obtained from the same plant were pooled. Two types of coinfestation experiments were carried out with different infestation regimes, using *T. urticae* Santpoort-2 (TuSP-2), *T. evansi* Viçosa-1 (TeV-1), and/or *T. urticae* DeLier-1 (TuDL-1). The first experiment consisted of six treatments, in which leaflets were infested with: no mites (control); 15 TuSP-2; 15 TeV-1; 30 TuSP-2; 30 TeV-1; or 15 TuSP-2 + 15 TeV-1 (coinfestation). Ten plants were used per treatment. The second experiment consisted of eight treatments: no mites; five TuSP-2; 15 TuSP-2; 15 TuDL-1; 25 TuDL-1; 15 TuSP-2 + 15 TuDL-1; five TuSP-2 + 15 TuDL-1; and five TuSP-2 + 25 TuDL-1. Six plants were used per treatment. This experiment was repeated twice.

### Isolation of phytohormones and analysis by means of LC-MS/MS

Phytohormone analysis was performed using the procedure of Wu *et al*. (2007) with some minor modifications (Methods S2; Table S1). Amounts were compared across treatments per time point independently using ANOVA with ‘spider mite strain’ as factor. Means of each group were compared by LSD *post hoc* test using PASW Statistics 17.0.

### Gene expression analysis

Total RNA was isolated as described in Verdonk *et al*. (2003). Two micrograms of DNAse-treated RNA was used for cDNA synthesis and 1 μl of 10-times-diluted cDNA served as a template for a 20 μl quantitative reverse-transcriptase polymerase chain reaction (qRT-PCR) using the Platinum SYBR Green qPCR-SuperMix-UDG kit (Invitrogen) and the ABI 7500 Real-Time PCR system (Applied Biosystems, Foster City, CA, USA). To survey tomato defenses, we analyzed expression of the following genes: *PPO-D*,*PPO-F*,*JIP-21*,*GAME-1*,*TD-2*,*THM27*,*LX*,*PR-1a*,*PR-P6*,*PI-IIc* and *PI-IIf*. *Actin* was used as a reference gene. PCR-generated amplicons were sequenced to verify primer specificity. Gene identifiers, primer sequences and references are listed in Table S2. The normalized expression (NE) data were calculated by the Δ*C*_t_ method 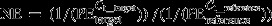; in which PE is the primer efficiency and *C*_t_ is the cycle threshold. The NE of each target gene was compared per time point independently using a nested ANOVA with ‘spider mite strain’ as factor and ‘technical replicate’ (i.e. two for each reaction) nested into the corresponding biological replicate (cDNA sample). Means of each group were compared by Fisher's LSD *post hoc* test using PASW Statistics 17.0. To plot the relative expression, NE values were scaled to the treatment with the lowest average NE.

## Results

### Selection of putative suppressor genotypes from natural populations

To identify and isolate putative JA defense-suppressing *T. urticae*, adult female spider mites were collected from natural populations found on three different host plants. We reasoned that the fecundity of JA-suppressor strains should be equally high on tomato (*S. lycopersicum*) WT and on the JA-biosynthesis mutant tomato *def-1*, as suppression will only be favored by natural selection when improving the reproductive performance of mites. Hence we tested the reproductive performance of each strain on these plants. *Tetranychus urticae* Santpoort-2 mites produced 34 ± 3 eggs on *def-1*, but only 22 ± 1 on WT (Fig. S4; Student's *t*-test: *P *=* *0.003), confirming its susceptibility to JA-mediated defenses (Kant *et al*., 2008). By contrast, mites from the putative suppressor strains *T. urticae* DeLier-1, *T. evansi* Viçosa-1, and Algarrobo-1 produced a similar number of eggs on both genotypes of plant (Student's *t*-test, *P *>* *0.05).

### The reproductive performance of defense-susceptible *T. urticae* Santpoort-2 mites increases when sharing a leaflet with the putative suppressor strains

**Fig 1:**
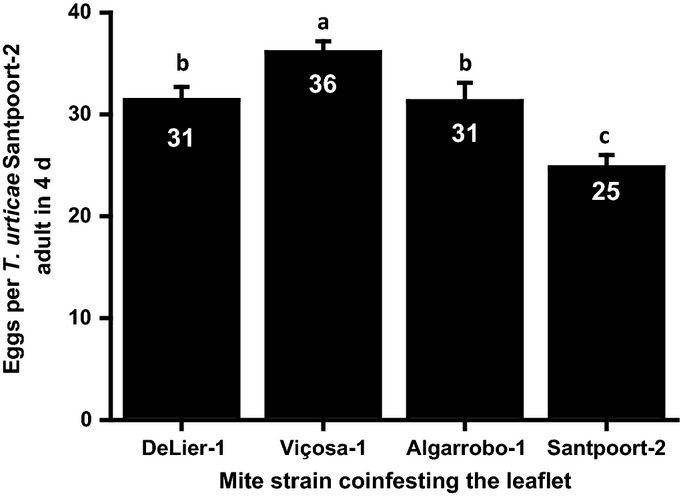
The reproductive performance of the jasmonic acid (JA)-defense-inducing and -susceptible *Tetranychus urticae* Santpoort-2 increases on tomato (*Solanum lycopersicum*) leaflets shared with spider mites from suppressor strains. The figure shows the average (+ SEM) number of eggs produced by adult female mites of strain *T. urticae* Santpoort-2 per 4 d on leaflets simultaneously coinfested with 15 adult females of *T. urticae* DeLier-1, *T. evansi* Viçosa-1, or *T. evansi* Algarrobo-1, or with *T. urticae* Santpoort-2 as a control. Numbers within the bars indicate the average egg production. Bars annotated with different letters were significantly different according to Fisher's least significant difference (LSD) test (*P* ≤ 0.05) after ANOVA.

Using the performance test on *def-1* and WT plants, we could not exclude the possibility that a high reproductive performance on WT plants is the result of direct resistance to induced tomato JA defenses. Hence we reasoned that only a genuine defense suppressor should be able to boost the reproductive performance of a defense-susceptible mite when both reside on the same leaflet. Indeed, all three strains clearly and significantly boosted the performance of *T. urticae* Santpoort-2 (Fig.1). Compared with the control, where *T. urticae* Santpoort-2 ‘shared a leaflet with itself’, *T. urticae* DeLier-1 and *T. evansi* Algarrobo-1 improved the susceptible strains’ fecundity with > 25%, while *T. evansi* Viçosa-1 did so with over 45%.

### The *T. evansi* strains suppress expression of tomato genes that mark JA, SA and senescence, but the suppressor *T. urticae* strain only suppresses that of genes induced late in the interaction

In order to narrow down the mechanisms that underlie the positive effect of putative suppressor strains on the fecundity of the susceptible strain, we assessed the magnitude and timing of defense-related phytohormone and transcript accumulation in response to each of the strains.

**Fig 2:**
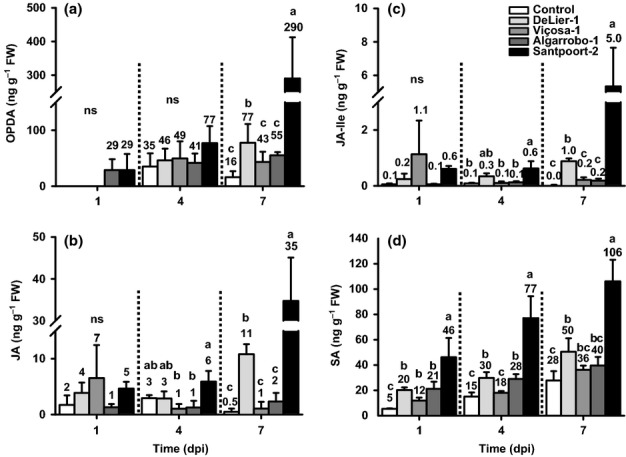
The amounts of 12-oxo-phytodienoic acid (OPDA), jasmonic acid (JA), jasmonic acid-isoleucine (JA-Ile), and free salicylic acid (SA) accumulated in spider mite-infested tomato (*Solanum lycopersicum*) leaflets during the course of 7 d. The figure shows the average (+ SEM) amounts of OPDA (a), JA (b), JA-Ile (c), and free SA (d) at 1, 4, and 7 d postinfestation (dpi) of tomato leaflets with 15 adult females from strain *Tetranychus urticae* DeLier-1, *T. evansi* Viçosa-1, *T. evansi* Algarrobo-1, or *T. urticae* Santpoort-2. Uninfested leaflets were used as controls. OPDA was not detected at 1 dpi in control, *T. urticae* DeLier-1, and *T. evansi* Viçosa-1 samples. Bars annotated with different letters were significantly different according to Fisher's least significant difference (LSD) test (*P* ≤ 0.05) after ANOVA. Bars marked with ‘ns’ did not test differently in the ANOVA. Data were log-transformed before the statistical analysis.

In general, *T. urticae* Santpoort-2 induced a significant increase of the oxylipins 12-oxo-phytodienoic acid (OPDA, the JA-precursor), JA, and JA-Ile at 7 dpi, with JA and JA-Ile already being significantly higher than uninfested controls at 4 dpi (Fig.2a–c). The accumulation of free SA upon infestation with *T. urticae* Santpoort-2 mites was even more rapid, that is, significantly higher than controls after 1 d, and appeared continuous (Fig.2d). Notably, the basal SA concentration in control plants increased as they grew older (Fig.2d: *F*_2,8_ = 6.010, *P *=* *0.025). Phytohormone accumulation induced by suppressor *T. urticae* DeLier-1 followed the same temporal pattern, albeit consistently at *c*. two to five times lower levels (Fig.2). Whereas concentrations of the defense-related phytohormones clearly peaked at 7 dpi with either of the two *T. urticae* strains, only minor, nonsignificant, increases were observed for any of these hormones after prolonged infestation with the *T. evansi* strains (Fig.2), even though they induced SA to concentrations similar to those induced by *T. urticae* DeLier-1 at 1 dpi.

We then selected 10 genes related to plant defenses for a detailed time-course expression analysis via qRT-PCR using the same samples. We selected *Polyphenol-oxidase-D* (*PPO-D*) and *PPO-F* (Newman *et al*., 1993; Thipyapong *et al*., 2004), *Glycoalkaloid metabolism-1* (*GAME-1*) (Itkin *et al*., 2011), the *AtMYB4/PhMYB4* homolog *THM27* (Mintz-Oron *et al*., 2008), the Cathepsin-D-inhibitor/chymotrypsin inhibitor encoding gene *Jasmonate-inducible Protein-21* (*JIP-21*) (Lisón *et al*., 2006), *Threonine Deaminase-2* (*TD-2*) (Gonzales-Vigil *et al*., 2011), the senescence-associated T2-like RNAse *ribonuclease LX* (*LX*) (Lers *et al*., 2006), *Pathogenesis-related protein 1a* (*PR-1a*) (Tornero *et al*., 1997), *PR-P6* (Van Kan *et al*., 1992), and *Proteinase Inhibitor IIc* (*PI-IIc*) (Gadea *et al*., 1996)

**Fig 3:**
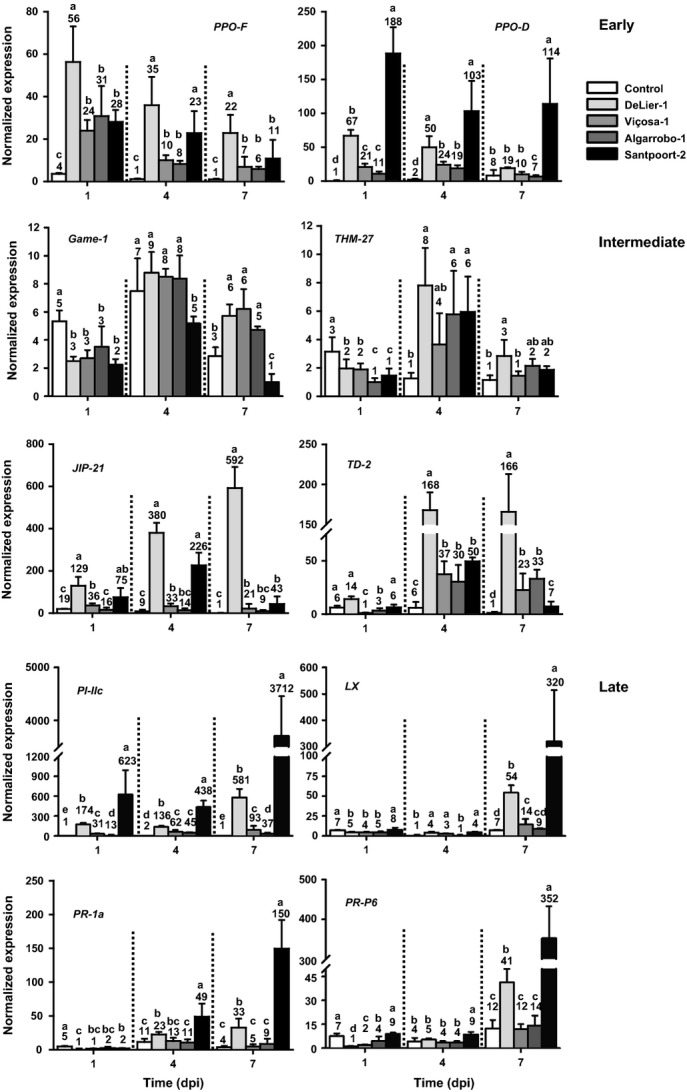
Relative transcript abundances of various defense-related genes in spider mite-infested tomato (*Solanum lycopersicum*) leaflets during the course of 7 d. Based on the *Tetranychus urticae* Santpoort-2 samples, the genes separated into three groups: those whose transcript abundances were highest at 1 d postinfestation (dpi) were annotated as ‘early’; those with a peak at 4 dpi as ‘intermediate’; and those with a peak at 7 dpi as ‘late’. Compared with *T. urticae* Santpoort-2, both *Tetranychus evansi* Viçosa-1 and *T. evansi* Algarrobo-1 mites suppressed genes from all three groups, while *T. urticae* DeLier-1 mites only moderately suppressed the ‘late’ defense genes. Uninfested leaflets were used as controls. The bars represent the means (+ SEM) of the normalized transcript abundances scaled to the lowest mean value per 7 d gene panel. Transcript abundances were normalized to actin. Numbers above the bars indicate the mean value represented by the bar. Expression data were statistically evaluated per day and bars annotated with different letters were significantly different according to Fisher's LSD test (*P* ≤ 0.05) after ANOVA. Gene identifiers and corresponding references can be found in Table S2. *GAME-1*,*Glycoalkaloid Metabolism-1*;*JIP-21*,*Jasmonate-inducible protein-21*;*LX*,*RNase Lycopersicon extravacuolar*;*PI-IIc*,*Proteinase Inhibitor IIc*;*PPO-D*,*Polyphenol-oxidase-D*;*PPO-F*,*Polyphenol-oxidase-F*;*PR-1a*,*Pathogenesis-related protein 1a*;*PR-P6*,*Pathogenesis-related protein 6*;*TD-2*,*Threonine Deaminase-2*;*THM27*,*Tomato Hypocotyl Myb 17*.

Using *T. urticae* Santpoort-2 as a benchmark, the expression pattern of the selected genes clustered into three groups (Fig.3, black bars): those with the highest transcript abundance at 1 dpi (referred to as ‘early’; *PPO-D* and *PPO-F*), 4 dpi (‘intermediate’; *GAME-1*;*THM27*;*JIP-21* and *TD-2*), or 7 dpi (‘late’; *LX*;*PI-IIc*;*PR-1a* and *PR-P6*). Except for *GAME-1*,*T. urticae* Santpoort-2 mites induced expression of all nine other genes in the tomato leaflets.

Rapid increased expression of the ‘early’ genes was evident after infestation with suppressor *T. urticae* DeLier-1 (Fig.3). Expression levels of *PPO-F* were even higher in the DeLier-1 samples than in the Santpoort-2 ones. This was also observed for the ‘intermediate’ genes *JIP-21* and *TD-2*. Moreover, contrary to Santpoort-2-infested leaflets, transcript abundances of all ‘intermediate’ genes in DeLier-1-infested leaflets remained above control values at 7 dpi. The expression patterns of the ‘late’ genes resulting from DeLier-1 and Santpoort-2 feeding, respectively, were similar, but in general DeLier-1 mites induced lower transcript abundances.

The two *T. evansi* suppressor strains both significantly induced the ‘early’ defense marker genes (Fig.3). Timing and magnitude of suppression and subsequent induction of *GAME-1* and *THM-27* by *T. evansi* were similar to that of the *T. urticae* DeLier-1 strain, but the levels of induction differed considerably for *JIP-21* and *TD-2*, as the *T. evansi* strains induced both genes only slightly after 4 and 7 d. When compared with levels induced by *T. urticae* Santpoort-2 at 7 dpi, *T. evansi* inhibited *JIP-21*, but induced *TD-2*. Of the ‘late’ genes, analogous to *T. urticae* DeLier-1, only *PI-IIc* (a JA marker gene; Fig. S5; Notes S2) was induced at 1 dpi, while the three other genes were suppressed. At later time points, transcript abundances of *PI-IIc* remained elevated, albeit to a far lower extent than with the *T. urticae* strains, and those of *LX* (senescence), *PR-1a*, and *PR-P6* (both SA markers) returned to control values, or slightly higher, that is, for *LX* after infestation with Viçosa-1 (Fig.3).

Based on these phytohormone and gene expression studies, we conclude that each mite strain triggers a unique defense response pattern in tomato leaflets, but that, at a particular time and compared with inducer *T. urticae* Santpoort-2, the two *T. evansi* strains suppressed ‘early’, ‘intermediate’, and ‘late’ genes, while the *T. urticae* suppressor strain DeLier-1 only suppressed the ‘late’ defense genes.

### Induction and suppression of defenses do not correlate with feeding intensity

We explored the mites’ feeding intensities as an alternative explanation for differences in the magnitude of defense induction. To do so, we assessed the total area of chlorotic lesions as a result of mite feeding (Kant *et al*., 2004). Notably, on leaflets infested with either of the suppressor mites, this typical white-on-green feeding damage pattern persisted at least until 7 dpi (Fig. S6a), while on leaves with *T. urticae* Santpoort-2 the lesions got increasingly surrounded by small areas of white-yellowish senescence and sometimes small oedema and russeting (Fig. S6b). To only assess feeding damage, we visually excluded these senesced areas as much as possible. The two *T. evansi* strains produced *c*. 100 mm^2^ of feeding damage (Fig. S6c), corresponding to *c*. 9% of the total leaflet area. The two *T. urticae* mite strains produced a total feeding damage of *c*. 40 mm^2^, corresponding to *c*. 3.5% of the leaflet area. The *T. evansi* strains thus inflicted at least twice as much feeding damage as the *T. urticae* strains. When including the senesced areas, damage inflicted by *T. urticae* Santpoort-2 equaled that of the *T. evansi* strains (data not shown). Hence, there was no positive relationship between the extent to which defenses were induced and the total leaf area that was damaged.

### *Tetranychus evansi* suppresses the *T. urticae*-induced expression of JA and SA marker genes but not the upstream accumulation of JA-Ile or SA

As the most clear-cut evidence for defense suppression by spider mites is demonstrated by the increased reproductive performance of the JA defense-susceptible *T. urticae* Santpoort-2 on coinfested leaflets (Fig.1), and differences in JA and SA defense-related phytohormone (Fig.2) and transcript abundances (Fig.3) between inducer and suppressor strains in ‘single strain-infested’ leaflets were most apparent at 7 dpi, we combined both experiments to determine whether suppressor mites still manage to inhibit these defense signaling pathways when sharing a leaflet with inducer Santpoort-2.

We first selected the most potent suppressor strain, *T. evansi* Viçosa-1 (Fig.1), and introduced 15 adult females to a leaflet to which, simultaneously, 15 adult *T. urticae* Santpoort-2 mites were also introduced. Seven days later we compared the concentrations of JA-Ile and SA plus expression levels of *PI-II*c and *PR-1a* from these coinfested leaflets with those of uninfested leaflets (negative control), as well as with leaflets with only 15 adult *T. urticae* Santpoort-2 mites or only 15 *T. evansi* Viçosa-1 mites (positive controls). Finally, infestations with only 30 adult *T. urticae* Santpoort-2 mites or only 30 *T. evansi* Viçosa-1 were included to control for density-dependent effects.

**Fig 4:**
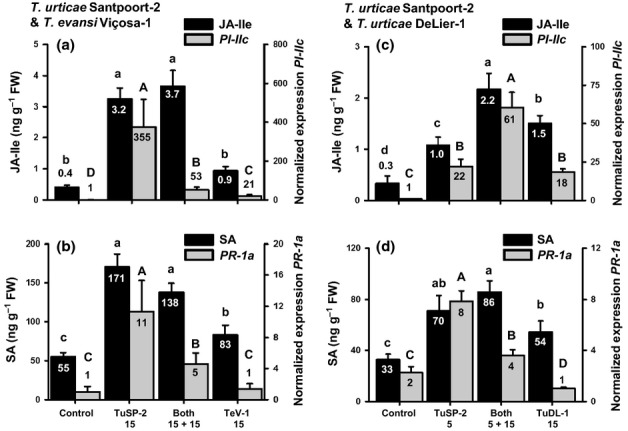
The amounts of jasmonic acid-isoleucine (JA-Ile) and salicylic acid (SA), along with transcript abundances of *Proteinase Inhibitor IIc* (*PI-IIc*) and *Pathogenesis-related 1a* (*PR-1a*) in tomato (*Solanum lycopersicum*) leaflets after 7 d of infestation with inducer *Tetranychus urticae* Santpoort-2, suppressor *T. evansi* Viçosa-1, and suppressor *T. urticae* DeLier-1, or a combination of inducer and either of the suppressors. The figure shows the amounts of JA-Ile and *PI-IIc* transcript (a, c) and the amounts of free SA and *PR-1a* transcript (b, d). Leaflets were infested with *T. urticae* Santpoort-2 (TuSP-2), *T. evansi* Viçosa-1 (TeV-1), or *T. urticae* DeLier-1 (TuDL-1), or simultaneously with TuSP-2 and either TeV-1 or TuDL-1 (both). Uninfested leaflets were used as controls. The numbers below the *x*-axis indicate the number of adult female mites used to infest the leaflets. The bars show the means (+ SEM), which are given as numbers within or above the bars. Transcript abundances were normalized to actin and scaled to the lowest mean per panel. Bars annotated with different letters (lowercase for JA-Ile and SA; uppercase for *PI-IIc* and *PR-1a*) were significantly different according to Fisher's least significant difference (LSD) test (*P* ≤ 0.05) after ANOVA.

In line with the previous results, infestation with 15 *T. urticae* Santpoort-2 mites resulted in strongly induced JA and SA defenses (Fig.4a,b). The plant's defense responses to Santpoort-2 mites increased in a density-dependent manner (Fig. S7). The 15 *T. evansi* Viçosa-1 mites caused minor increases in JA-Ile and SA concentrations, but higher densities of *T. evansi* Viçosa-1 did not further elevate hormone concentrations or *PI-IIc* expression, while even lowering that of *PR-1a* (Fig. S7).

In leaflets coinfested with 15 *T. urticae* Santpoort-2 mites and 15 *T. evansi* Viçosa-1, concentrations of JA-Ile and SA were equal to those only infested with Santpoort-2 (Fig.4a,b). However, expression levels of *PI-IIc* and *PR-1a* were intermediate, that is, significantly lower than in leaflets with 15 *T. urticae* Santpoort-2, but still higher than in the leaflets with 15 *T. evansi* Viçosa-1 mites. Taken together, in coinfested leaflets, *T. evansi* Viçosa-1 does not suppress phytohormone accumulation, but only the expression of the downstream marker genes. Hence, suppression by *T. evansi* Viçosa-1 probably occurs downstream of phytohormone accumulation.

To test whether defense suppression within *T. urticae* species operates in the same way, we repeated the coinfestation experiment with *T. urticae* DeLier-1 as the suppressor. Here we used only five *T. urticae* Santpoort-2 mites, as the magnitude of suppression by DeLier-1 appeared lower than that of the *T. evansi* strains (Figs3). The tomato JA-defense response induced by the two *T. urticae* strains together appeared to be additive (Figs4c,d,S8). By contrast, the SA concentrations of coinfested leaflets equaled those infested only with Santpoort-2 and the *PR-1a* transcript abundances were suppressed down to intermediate values by DeLier-1 (Fig.4c,d). Using 15 instead of five Santpoort-2 mites, or when using 25 DeLier-1 individuals, we did not observe significant cosuppression of defenses (Fig. S8). This indicates that DeLier-1 is a less potent suppressor of SA defenses than *T. evansi* Viçosa-1 and, although it induced a significantly lower JA response even at higher densities than Santpoort-2, it is unable to significantly suppress the Santpoort-2-induced JA-defense response.

## Discussion

Suppression of plant immunity is especially well known from plant pathogens (Abramovitch *et al*., 2006; Burgyán & Havelda, 2011; De Jonge *et al*., 2011) and nematodes (Haegeman *et al*., 2012). In recent years, some herbivorous insects were also found to suppress plant defenses (Hogenhout & Bos, 2011), but defense suppression by Chelicerates is still poorly documented (Kant *et al*., 2008; Sarmento *et al*., 2011a). Hence, we have characterized three JA defense-suppressing spider mite strains for the extent to which they are able to lower tomato defenses and to promote the reproductive performance of a JA defense-sensitive competing mite strain. We showed that *T. urticae* DeLier-1 is a moderate suppressor of induced defenses that improves the reproductive performance of competing Santpoort-2 mites by 25%. Furthermore, we showed that suppression by the strains *T. evansi* Viçosa-1 and Algarrobo-1 inhibits JA and SA responses simultaneously and, hence, is not depending on the JA–SA antagonism. Moreover, suppression by *T. evansi* Viçosa-1 most likely occurs downstream of phytohormone accumulation and is powerful enough to cosuppress the expression of defense genes induced by *T. urticae* Santpoort-2, thereby boosting the reproductive performance of its competitor by 45%.

Induction of JA defenses by *T. urticae* Santpoort-2 parallels induction of SA defenses, while, the other way around, suppression of JA defenses by the other three mite strains parallels suppression of SA defenses (Figs3). In fact, in tomato leaflets coinfested with *T. urticae* Santpoort-2 and *T. evansi* Viçosa-1 mites, *PI-IIc* (JA-defense marker) and *PR-1a* (SA-defense marker) were both suppressed, even though JA-Ile and SA were induced to concentrations found in leaflets exclusively infested with Santpoort-2 (Fig.4a,b). We therefore conclude that defense suppression by these spider mites acts downstream of phytohormones and independent of the JA–SA antagonism. By contrast, *T. kanzawai* (Ozawa *et al*., 2011) and some other herbivores (Zarate *et al*., 2007; Weech *et al*., 2008; Chung *et al*., 2013) were suggested to manipulate the JA–SA crosstalk mechanism to suppress JA defenses.

The low concentrations of phytohormones detected in leaflets infested with only suppressor mites thus do not seem to be the cause of suppression of downstream defenses, but rather are a consequence, possibly as a result of altered feedback regulation of hormone biosynthesis (Chini *et al*., 2007; Paschold *et al*., 2007 Serrano *et al*., 2013). The question remains as to why the simultaneous SA and JA responses induced by Santpoort-2 mites do not antagonize each other? One explanation is that these responses might be heterogeneous in space, for example, one may dominate at the feeding site and the other in surrounding tissues. Consequently, by harvesting complete leaflets we mix what in reality is an SA/JA response mosaic. Indeed, in wounded *Nicotiana attenuata* (Schittko *et al*., 2000; Wu *et al*., 2007), *Pseudomonas*-infected *Phaseolus vulgaris* (Meier *et al*., 1993) and elicitor-treated *N. tabacum* (Dorey *et al*., 1998) and *Zea mays* (Engelberth *et al*., 2012), defense responses were found to be stronger close to the wounded area or infection site. Not all defenses follow this pattern, as *PI-I* transcript abundances were found to be highest distant to the wound site (Howe *et al*., 1996). Another explanation might be that simultaneous SA and JA responses actually do antagonize each other and what we observe are intermediate responses, as was also suggested to happen in *N. attenuata* infested with *Manduca sexta* (Diezel *et al*., 2009). Thus, although JA and SA may crosstalk during induction of defenses by mites, their antagonistic interaction is not involved in defense suppression by mites.

Results from coinfestation experiments of Santpoort-2 and DeLier-1 mites suggest that defense suppression by DeLier-1 functionally operates in the same way. The mechanistic evidence, though, is complex, as DeLier-1 triggers a defense response that is clearly distinct from that of the *T. evansi* strains. The phytohormone accumulation data (Fig.2) and the expression data on the ‘late’ defense genes (Fig.3) suggest that DeLier-1 may delay defenses rather than fully block them. Despite the strong and fast induction of several JA-regulated defense genes, for example, *PPO-F*,*JIP-21*, and *PI-IIc* (Fig.3), suppression of JA-mediated defenses by DeLier-1 was shown to occur within the first 4 d of infestation (Fig. S3a). However, after 7 d of coinfestation with inducer mites, suppression was clear for *PR-1a*, but not for *PI-IIc* (Fig.4c,d). Moreover, this suppressive effect on *PR-1a* was only observed when DeLier-1 outnumbered the inducer mites three to one, which confirms it is a less potent suppressor than Viçosa-1 (Fig.1). Together, this suggests we may be overlooking the (more) relevant defenses and/or that SA defenses play a more important role in the defense response against mites than they do against herbivorous insects.

The defense suppression we observed does not act on the expression of all defense genes in a similar manner and magnitude (Fig.3). Both *PPO* genes were strongly and rapidly induced by all mite strains, including the suppressors, hence their classification as ‘early’. For *PPO-F*,*T. urticae*, DeLier-1 even induced the overall highest transcription. PPOs are believed to act in the guts of herbivores where they may convert plant-derived flavonoids into quinones. These are highly reactive molecules that can make amino acids indigestible, can damage gut enzymes or DNA, and can form reactive oxygen species (Constabel & Barbehenn, 2008).

Two of the ‘intermediate’ response genes, *GAME-1* and *THM27*, are involved in regulation of the secondary metabolism, that is, the alkaloid and flavonoid metabolism, respectively. The same temporal (bell-shaped) expression pattern was observed for both genes in all mite-infested leaflets. *GAME-1* is involved in the glycosylation of steroidal alkaloids, in particular aglycon tomatidine, presumably to reduce the autotoxicity of these metabolites (Itkin *et al*., 2011, 2013). The gene was down-regulated in all leaflets at 1 dpi but remained down-regulated only in *T. urticae* Santpoort-2-infested leaflets. Tomatidine was found to be toxic to root-knot nematodes and, while most insects can cope with it, the potato aphid suffers from high concentrations (Milner *et al*., 2011). Hence, whether down-regulation of *GAME-1* in *T. urticae* Santpoort-2 infested leaflets reflects an effective defense response remains to be determined.

THM27 is an R2R3-MYB transcription factor that controls flavonoid metabolism (Adato *et al*., 2009; Dal Cin *et al*., 2011) and is homologous to *AtMYB4* (Mintz-Oron *et al*., 2008) and *PhMYB4* (Colquhoun *et al*., 2011). All mite strains down-regulated *THM27* at 1 dpi, albeit not all significantly. At 4 dpi, however, it was significantly up-regulated, after which expression levels reduced again.

To put this into perspective, tomato plants might up-regulate the biosynthesis of lignins and flavonoids, including PPO substrates (Constabel & Barbehenn, 2008), especially early in the interaction, but then switch to alternative measures when the infestation progresses. Expression of *JIP-21*,*TD-2*, and *PI-IIc* might be part of such alternative measures. They encode enzymes thought to interfere with the herbivore's digestive processes (Chen *et al*., 2005; Lisón *et al*., 2006; Gonzales-Vigil *et al*., 2011) and were induced at 4 and/or 7 dpi by all mite strains, although in a nonuniform way. For instance, after 7 d of infestation, suppressor mites had induced *TD-2* to higher levels than did Santpoort-2, while this pattern was reversed for *PI-IIc*. Some of the PR genes, which belong to a different class of defense genes (Van Loon & Van Strien, 1999), were sometimes found to be up-regulated upon infestation with DeLier-1, but never by the *T. evansi* strains.

Using marker genes for drawing accurate conclusions regarding complex processes strongly depends on the correlation between the expression levels of such genes and the associated process. When investigating the correlation between the ‘classical’ tomato JA-marker gene *PI-IIf* (Notes S2) and JA concentrations, we noticed that, especially at low JA concentrations, the gene was regularly highly expressed (Fig. S5). This suggests that not only JA but also other (hormonal) signals activated by spider mites influence its regulation. However, the correlation between the expression of another family member of this gene (*PI-IIc*) and JA concentrations was much stronger and hence we used this gene as a marker for JA-related processes induced by spider mites. This underpins the fact that marker genes may require context-specific validation before being used as process indicators.

In summary, each mite strain affects the expression of tomato defense genes differently, but the putative negative effect of each of these genes on the spider mite performance remains largely unknown and is subject to future research. Furthermore, the expression pattern of the senescence-marker *LX* (Lers *et al*., 2006) at 7 dpi perfectly reflected the visual development of senescence in the infested leaflets. Possibly as a result of the induced defenses, leaflets infested with Santpoort-2 went into senescence early and in a density-dependent way before they died, while senescence in leaflets infested with DeLier-1 was less severe and came days later and *T. evansi*-infested leaflets dried out without showing clear signs of senescence before dying.

The mechanism by which spider mites suppress host defenses is still unclear. Some phytopathogens, vectored by arthropods, have been implicated in the suppression of plant defenses, putatively to (indirectly) enhance their own fitness (Belliure *et al*., 2005; Sugio *et al*., 2011; Casteel *et al*., 2012, 2014; Zhang *et al*., 2012; Chung *et al*., 2013). Preliminary data, though, indicate that spider mite-associated microbes do not mediate suppression of plant defenses (data not shown). Analogous to phytopathogens (Da Cunha *et al*., 2007; De Jonge *et al*., 2011), aphids (Rodriguez & Bos, 2013), and nematodes (Haegeman *et al*., 2012), spider mites may also secrete effectors via their saliva into plant tissues to interfere with host immune responses (Alba *et al*., 2011). The spider mite genome (Grbić *et al*., 2011) encodes at least 293 putative salivary proteins (with an *E*-value < 1E–20), and thus mites are likely to secrete a rich cocktail of proteins while feeding. Whether (some of) these predicted salivary proteins truly are involved in suppression (and/or induction) of plant defenses – and what their *in planta* targets are – remains to be demonstrated. Finally, the concurrent suppression of JA and SA defenses hints at manipulation of the redox homeostasis (Koornneef *et al*., 2008; Gruner *et al*., 2013), but as inhibition of phytohormone accumulation appears nonessential for suppression of the downstream response, other mechanisms are likely to be at play as well.

Our data suggest that defense-suppression traits are not very rare in natural populations of spider mites, especially not for *T. evansi*. Judging the effects that suppressors have on tomato, these traits may be diverse across and within species and be intertwined with (unrelated) traits that cause induction of plant defenses (Kant *et al*., 2008). However, the ecological costs and benefits of defense suppression are still unclear. Rationally, resistance (Kant *et al*., 2008) seems a ‘safer’ trait than the ability to suppress, as suppression can clearly benefit competing species as well (Fig.1). We found putative suppressor genotypes within all three *T. urticae* populations we sampled (five putative suppressor strains among the 239 strains tested). This suggests that the trait is either maintained by frequency-dependent selection or results from genetic drift. Given the observation that suppression increases the fitness of these mites in the absence of competitors while – potentially – decreasing it in their presence suggests that competitor-associated fluctuating selection may be a driving force. By contrast, both *T. evansi* haplotypes suppressed defenses similarly and we did not observe intraspecific variation, suggesting that for this species the suppression trait got to fixation. The natural host range of the *T. evansi* haplotype from the Brazilian clade (such as *T. evansi* Viçosa-1; Fig. S2a) appears to be narrower than the ones from the Spanish clade (such as *T. evansi* Algarrobo-1; Fig. S2a), but both haplotypes are frequently found on several of the same solanaceous species as *T. urticae* in the same geographical regions (Navajas *et al*., 2013). Given our observation that *T. urticae* can increase its reproductive performance up to 45% when sharing a leaflet with *T. evansi* under laboratory conditions, we would not expect the displacement of natural *T. urticae* populations by *T. evansi* as is currently taking place on several host plants in southern Europe (Ferragut *et al*., 2013). Hence, the key question is how defense-suppressing herbivores manage to prevent or overcome the negative effects such that they themselves receive the largest net benefit from the manipulation? One of the answers may be that *T. evansi* monopolizes its feeding site by the production of extraordinarily large quantities of silken web, which not only shields the population from acaricides and natural enemies but also makes it hard for competing tetranychid mites to invade (Lemos *et al*., 2010; Sarmento *et al*., 2011b). Although speculative, this trait may have been selected under pressure of competitors facilitated by the suppressed defenses. Interestingly, *T. urticae* DeLier-1 mites do not produce excessive amounts of webbing but do promote the reproductive performance of *T. urticae* Santpoort-2 and, thus, if and how moderate plant-defense suppressors such as DeLier-1 protect their manipulated resources from competitors warrant more in-depth ecological research.

## Supporting Information

**Fig. S1**Photos of adult female spider mites from each of the four strains used for this study. **Fig. S2** Phylogenetic trees based upon the *cytochrome oxidase subunit 1* (*COI*) sequences from spider mites, including sequences from the mite strains used for this study. **Fig. S3** Fecundity of putative JA defense-suppressing *T. urticae* strains on *def-1*, wild-type (WT) and *35S::Prosystemin* tomato and induction of *Proteinase Inhibitor IIf* (*PI-IIf*) by these strains upon infestation of WT plants. **Fig. S4** Reproductive performance of adult female spider mites on wild-type and *def-1* tomato. **Fig. S5** Regression analysis of jasmonic acid (JA) content and expression levels of *Proteinase Inhibitor IIc* (*PI-IIc*) and *PI-IIf* upon infestation of tomato leaflets with spider mites. **Fig. S6** Feeding damage inflicted by adult female spider mites on tomato leaflets. **Fig. S7** Concentrations of jasmonic acid-isoleucine (JA-Ile) and salicylic acid (SA), plus transcript abundances of *Proteinase Inhibitor IIc* (*PI-IIc*) and *Pathogenesis-related protein 1a* (*PR-1a*) in tomato leaflets after 7 d of infestation with spider mites from inducer strain *T. urticae* Santpoort-2, suppressor *T. evansi* Viçosa-1 or both strains together. **Fig. S8** Concentrations of jasmonic acid-isoleucine (JA-Ile) and salicylic acid (SA), plus transcript abundances of *Proteinase Inhibitor IIc* (*PI-IIc*) and *Pathogenesis-related protein 1a* (*PR-1a*) in tomato leaflets after 7 d of infestation with spider mites from inducer strain *T. urticae* Santpoort-2, suppressor *T. urticae* DeLier-1, or both strains together. **Table S1** Parameters used for detection of phytohormones by liquid chromatography-mass spectrometry/mass spectrometry (LC-MS/MS) **Table S2** qRT-PCR primer sequences **Methods S1** Protocol for infestation of tomato plants with spider mites. **Methods S2** Protocol for the extraction and quantification of phytohormones from tomato leaves. **Notes S1** Sampling and rearing of spider mites. **Notes S2** The *Proteinase Inhibitor II* (*PI-II*) gene family in tomato.
