# Supplementary material for: Spider mites suppress tomato defenses downstream of jasmonate and salicylate independently of hormonal crosstalk
Source: New Phytol. 2014 Oct 8;205(2):828–40. doi: 10.1111/nph.13075 (PMC4301184; doi:10.1111/nph.13075)
Supplement: Supplementary file 1 — Fig. S1Photos of adult female spider mites from each of the four strains used for this study. Fig. S2 Phylogenetic trees based upon the cytochrome oxidase subunit 1 (COI) sequences from spider mites, including sequences from the mite strains used for this study. Fig. S3 Fecundity of putative JA defense-suppressing T. urticae strains on def-1, wild-type (WT) and 35S::Prosystemin tomato and induction of Proteinase Inhibitor IIf (PI-IIf) by these strains upon infestation of WT plants. Fig. S4 Reproductive performance of adult female spider mites on wild-type and def-1 tomato. Fig. S5 Regression analysis of jasmonic acid (JA) content and expression levels of Proteinase Inhibitor IIc (PI-IIc) and PI-IIf upon infestation of tomato leaflets with spider mites. Fig. S6 Feeding damage inflicted by adult female spider mites on tomato leaflets. Fig. S7 Concentrations of jasmonic acid-isoleucine (JA-Ile) and salicylic acid (SA), plus transcript abundances of Proteinase Inhibitor IIc (PI-IIc) and Pathogenesis-related protein 1a (PR-1a) in tomato leaflets after 7 d of infestation with spider mites from inducer strain T. urticae Santpoort-2, suppressor T. evansi Viçosa-1 or both strains together. Fig. S8 Concentrations of jasmonic acid-isoleucine (JA-Ile) and salicylic acid (SA), plus transcript abundances of Proteinase Inhibitor IIc (PI-IIc) and Pathogenesis-related protein 1a (PR-1a) in tomato leaflets after 7 d of infestation with spider mites from inducer strain T. urticae Santpoort-2, suppressor T. urticae DeLier-1, or both strains together. Table S1 Parameters used for detection of phytohormones by liquid chromatography-mass spectrometry/mass spectrometry (LC-MS/MS) Table S2 qRT-PCR primer sequences Methods S1 Protocol for infestation of tomato plants with spider mites. Methods S2 Protocol for the extraction and quantification of phytohormones from tomato leaves. Notes S1 Sampling and rearing of spider mites. Notes S2 The Proteinase Inhibitor II (PI-II) gene family in tomato. [file nph0205-0828-sd1.docx]

## Supporting Information Figs S1–S8, Tables S1 & S2, Methods S1 & S2 and Notes S1 & S2

**Article title:** Spider mites suppress tomato defenses downstream of jasmonate and salicylate independently from hormonal crosstalk

**Authors:** Juan M. Alba^1*^, Bernardus C. J. Schimmel^1*^, Joris J. Glas^1^, Livia M. S. Ataide^2^, Maria L. Pappas^3^, Carlos A. Villarroel^4^, Robert C. Schuurink^4^, Maurice W. Sabelis^1^, Merijn R. Kant^1^

^1^Dept. of Population Biology, Institute for Biodiversity and Ecosystem Dynamics, University of Amsterdam, PO Box 94240, 1090 GE Amsterdam, the Netherlands; ^2^Dept. of Entomology, University of Viçosa, Viçosa, Minas Gerais, Brazil; ^3^Dept. of Agricultural Development, Laboratory of Agricultural Entomology and Zoology, Democritus University of Thrace, Pantazidou 193, 68 200, Orestiada, Greece; ^4^Dept. of Plant Physiology, Swammerdam Institute of Life Sciences, University of Amsterdam, PO box 94215, 1090 GE, Amsterdam, the Netherlands

* These authors contributed equally to the paper.

Author for correspondence: email: [m.kant@uva.nl](mailto:m.kant@uva.nl); phone: (+31)-20-5257793

**Fig. S1** Adult female spider mites (*Tetranychus* spp.) from each of the four strains used for this study. (a) *T. evansi* Viçosa-1 with two eggs; (b) *T. evansi* Algarrobo-1; (c) *T. urticae* DeLier-1; and (d) *T. urticae* Santpoort-2. Bar, 0.5 mm.

**Fig. S2** Maximum likelihood tree of the genus *Tetranychus* based upon mitochondrial *cytochrome oxidase subunit 1* (*COI*) sequences. Phylogenetic reconstruction based on the Neighbor-Joining method of the *COI* sequences of (a) the *T. evansi* strains and (b) the *T. urticae* strains used for this study. Codes in the tree refer to GenBank accessions. Numbers at nodes indicate bootstrap values. Nucleotide divergence was estimated by the Kimura 2-parameter model. The *T. urticae* Houten-1 *COI* sequence (GB: KF447572) was used as outgroup in the *T. evansi* phylogenetic tree. The *T. evansi* Viçosa-1 *COI* sequence (GB: KF447575) was used as outgroup in the *T. urticae* phylogenetic tree. Evolutionary distance of 0.01 is indicated by the scale bar below each tree. The phylogenetic analysis was performed using MEGA version 5 (Tamura *et al*. 2011). The species identity of *T. urticae* and *T. evansi* was confirmed on the basis of the aedeagus (the male reproductive organ) morphology (not shown).

**Fig. S3** Fecundity of adult females from each of the putative suppressor strains and *Tetranychus urticae* Santpoort-2 on *defenseless-1* (*def-1*), wild type (WT) and *35S::Prosystemin* (PS) tomato (*Solanum lycopersicum* cv Castlemart) and induction of *Proteinase Inhibitor IIf* (*PI-IIf*) by these strains upon infestation of WT plants. (a) Average (+ SEM) number of eggs produced by adult female mites of the putative suppressor strains and negative control *T. urticae* Santpoort-2 per 4 d on *def-1*, WT and *35S::Prosystemin*. Suppressors of induced JA-defenses produce an equal number of eggs on WT and *def-1*, but their oviposition rate is reduced on *35S::Prosystemin*, since the exceptionally strong JA-defenses employed by these plants cannot be suppressed by mites (Kant *et al*., 2008). (b) Relative transcript levels (mean + SEM) of *PI-IIf* in tomato leaflets after 4 d of infestation with 15 adult females from each of the putative suppressor strains. Uninfested- and *T. urticae* Santpoort-2-infested plants were used as negative and positive controls, respectively. Transcript levels were normalized to actin and scaled to the treatment with the lowest average normalized expression. The names of the putative suppressor strains as well as the host plant they were sampled from, i.e. *Euonymus europaeus*, *Lamium album*, or *Ricinus communis*, are indicated. Bars annotated with different letters were significantly different according to Fisher’s LSD test (*P* < 0.05) after ANOVA.

**Fig. S4** Reproductive performance of adult female spider mites on wild type and *defenseless-1* (*def-1*) tomato (*Solanum lycopersicum* cv Castlemart) plants. The figure shows the average number of eggs (+ SEM) produced by adult female mites of strains *Tetranychus urticae* DeLier-1, *T. evansi* Viçosa-1, *T. evansi* Algarrobo-1 and *T. urticae* Santpoort-2 per 4 d on wild type and *def-1* tomato leaflets. Numbers within the bars indicate the average egg production. Reproductive performance on wild type vs *def-1* was evaluated by means of a Student’s t-test: **, *P* < 0.01; ns, no significant difference was found (*P* > 0.05).

**Fig. S5** Regression between the jasmonic acid (JA) content and transcript levels of two JA marker genes, i.e. *Proteinase Inhibitor IIc* (*PI-IIc*) and *PI-IIf*, obtained from the same spider mite-infested tomato (*Solanum lycopersicum*) leaflets. The figure shows a scatter plot and trendline of JA-levels in nanogram (ng) per gram fresh leaf weight (gFW) vs (a, c, e, g) Log(*PI-IIc*) transcript levels or (b, d, f, h) vs Log(*PI-IIf*) transcript levels measured in leaflets 7 d post infestation with 15 adult (a, b) *Tetranychus urticae* Santpoort-2; (c, d) *T. urticae* DeLier-1; (e, f) *T. evansi* Viçosa-1; or (g, h) *T. evansi* Algarrobo-1. Transcript levels (NE) were normalized to actin. The regression analysis was performed on all possible scale combinations of dependent/independent variables and shown are the scale combinations with the best fit between observed and predicted values, i.e. having the highest coefficient of determination (R^2^). Each regression was tested by ANOVA (Sokal and Rohlf, 2012) and *P*-values (P) are presented within each plot.

**Fig. S6** Feeding damage on tomato (*Solanum lycopersicum*) leaflets inflicted by adult female spider mites from each of the suppressor strains and *Tetranychus urticae* Santpoort-2. The lacerate-and-flush feeding on mesophyll cells by suppressor mites and inducer *T. urticae* Santpoort-2 results in distinct damage phenotypes. (a) Speckled chlorotic lesions are characteristic for all mite species. (b) However, after prolonged feeding by *T. urticae* Santpoort-2 the lesions get increasingly surrounded by areas of white-yellowish senescence and micro-oedema, but not on plants infested with suppressor strains. (c) Total feeding damage on tomato leaflets produced by 15 adult female mites after 7 d of infestation. Bars represent the means (+ SEM), which are given as numbers within the bars. Bars annotated with different letters were significantly different according to Fisher’s LSD test (*P* < 0.05) after ANOVA. Bars (a, b), 1 mm.

**Fig. S7** The amounts of JA-Ile and SA, along with transcript levels of *PI-IIc* and *PR-1a* in tomato (*Solanum lycopersicum*) leaflets after 7 d of infestation with inducer *Tetranychus urticae* Santpoort-2, suppressor *T. evansi* Viçosa-1, or both strains together. The figure shows (a) the levels of jasmonic acid-isoleucine (JA-Ile) and *Proteinase Inhibitor IIc* (*PI-IIc*) transcript and (b) the levels of free salicylic acid (SA) and *Pathogenisis-related 1a* (*PR-1a*) transcript. Leaflets were infested with *T. urticae* Santpoort-2 (TuSP-2), *T. evansi* Viçosa-1 (TeV-1) or simultaneously with TuSP-2 + TeV-1 (both). Uninfested leaflets were used as controls. The numbers below the *x*-axis indicate the number of adult female mites used to infest the leaflets with. The bars show the mean (+ SEM) which are given as numbers within or above the bars. Phytohormone amounts are presented as nanogram (ng) per gram fresh leaf weight (gFW). Transcript levels were normalized to actin and scaled to the lowest mean per panel. Bars annotated with different letters (lowercase for JA-Ile and SA; uppercase for *PI-IIc* and *PR-1a*) were significantly different according to Fisher’s LSD test (*P* < 0.05) after ANOVA. The boxed data is also presented in Fig. 4a, b.

**Fig. S8** The amounts of JA-Ile and SA, along with transcript levels of *PI-IIc* and *PR-1a* in tomato (*Solanum lycopersicum*) leaflets after 7 d of infestation with inducer *Tetranychus urticae* Santpoort-2, suppressor *T. urticae* DeLier-1, or both strains together. The figure shows (a) the levels of jasmonic acid-isoleucine (JA-Ile) and *Proteinase Inhibitor IIc* (*PI-IIc*) transcript and (b) the levels of free salicylic acid (SA) and *Pathogenisis-related 1a* (*PR-1a*) transcript. Leaflets were infested with *T. urticae* Santpoort-2 (TuSP-2), *T. urticae* DeLier-1 (TuDL-1) or simultaneously with TuSP-2 + TuDL-1 (both). Uninfested leaflets were used as controls. The numbers below the *x*-axis indicate the number of adult female mites used to infest the leaflets with. The bars show the mean (+ SEM) which are given as numbers above the bars. Phytohormone amounts are presented as nanogram (ng) per gram fresh leaf weight (gFW). Transcript levels were normalized to actin and scaled to the lowest mean per panel. Bars annotated with different letters (lowercase for JA-Ile and SA; uppercase for *PI-IIc* and *PR-1a*) were significantly different according to Fisher’s LSD test (*P* < 0.05) after ANOVA. The boxed data is also presented in Fig. 4c, d.

**Table S1** Parameters used for detection of phytohormones and related compounds by LC-MS/MS

| Compound | Capillary  CID^1^  (V) | Molecular ion [M-H] (*m/z*) | Fragment ion  (*m/z*) | CE^2^  (V) | Reference |
| --- | --- | --- | --- | --- | --- |
| OPDA | -35 | 291 | 165 | 18 | Koo *et al*. (2009) |
| JA | -35 | 209 | 59 | 12 | Wu *et al*. (2007) |
| D_5_-JA (IS) | -35 | 213 | 61 | 12 | this paper |
| JA-Ile | -35 | 322 | 130 | 19 | Wu *et al*. (2007) |
| SA | -35 | 137 | 93 | 15 | Wu *et al*. (2007) |
| D_6_-SA (IS) | -35 | 141 | 97 | 15 | this paper |

^1^collision-induced dissociation; ^2^collision energy; IS, internal standard.

**Table S2** Nucleotide sequence of primers used for qRT-PCR analysis

| Target Gene | Name | GenBank (GB)  accession | Gen Model ITAG2.3: | Forward Primer  5’ 🡪 3’ | Reverse Primer  5’ 🡪 3’ | References |
| --- | --- | --- | --- | --- | --- | --- |
| *PPO-D* | *Polyphenol-oxidase-D* | Z12836.1 | Solyc08g074680.2.1 | GCCCAATGGAGCCATATC | ACATTCGATCCACATTGCTG | Newman *et al*. (1993) |
| *PPO-F* | *Polyphenol-oxidase-F* | AK247126.1 | Solyc08g074630.1.1 | CGGAGTTTGCAGGGAGTTATAC | TTGATCTCCACACTTTCAATGG | Newman *et al*. (1993) |
| *JIP-21* | *Jasmonate-inducible protein 21* | AJ295638.1 | Solyc03g098790.1.1 | ACTCGTCCTGTGCTTTGTCC | CCCAAGAGGATTTTCGTTGA | Lisón *et al*. (2006) |
| *GAME-1* | *Glycoalkaloid metabolism-1* | NM_001246924.1 | Solyc07g043490.1.1 | GCATTTTGGTCCGCTCTCTC | GCGCATTCAACCAATCTACAAC | Itkin *et al*. (2011) |
| *TD2* | *Threonine Deaminase-2* | M61915.1 | Solyc09g008670.2.1 | TGCCGTTAAAAATGTCACCA | ACTGGCGATGCCAAAATATC | Chen *et al*. (2005) |
| *THM27* | *Tomato Hypocotyl Myb 27* | NM_001247046.1 | Solyc10g055410.1.1 | AAAGTTGTCGTCTCCGATGG | TGTTTCCAAGGAGGCTATGG | Lin *et al*. (1996) |
| *LX* | *RNase Lycopersicon extravacuolar* | X79338.1 | Solyc05g007940.2.1 | GCGTCTTATTGCGACACGAGG | TGGAAATAGGCATGTTGGTTAAGAGC | Lers *et al*. (2006) |
| *PR-1a* | *Pathogenesis-related protein 1a* | AJ011520 | Solyc09g007010.1.1 | TGGTGGTTCATTTCTTGCAACTAC | ATCAATCCGATCCACTTATCATTTTA | Van Kan *et al*. (1992) |
| *PR-P6* | *Pathogenesis-related protein P6* | M69248.1 | Solyc00g174340.1.1 | GTACTGCATCTTCTTGTTTCCA | TAGATAAGTGCTTGATGTCCA | Van Kan *et al*. (1992) |
| *PI-IIc* | *Proteinase Inhibitor IIc* | X94946.1 | Solyc03g020050.2.1 | CAGGATGTACGACGTGTTGC | GAGTTTGCAACCCTCTCCTG | Gadea *et al*. (1996) |
| *PI-IIf* | *Proteinase Inhibitor IIf* | AY129402.1 | Solyc03g020080.2.1 | GACAAGGTACTAGTAATCAATTATCC | GGGCATATCCCGAACCCAAGA | Graham *et al*. (1985) |
| *Actin* | *Actin* | XM_004235020.1 | Solyc03g078400.2.1 | TTAGCACCTTCCAGCAGATGT | AACAGACAGGACACTCGCACT | Tomato Genome Consortium (2012) |

**Methods S1** Protocol for infestation of tomato plants with spider mites.

Unless otherwise indicated, we followed a standardized protocol for plant infestation experiments. We used adult female spider mites all of which within 48 h of the same age obtained from a so-called ‘egg-wave’. This egg-wave was generated by allowing random adult females from a base colony to produce eggs for 48 h on detached *Phaseolus vulgaris* cv Speedy (for *T. urticae*) or *Solanum lycopersicum* cv Castlemart (for *T. evansi*) leaflets which had been put flat on wet cotton wool, after which the adults are removed and the eggs allowed to hatch and mature in a climate room (25^o^C, 16 h : 8 h, light : dark, 60% RH, 300 µmol m^-2^ s^-1^). After 16 d, the 3 ± 1-d-old adult females were collected and transferred to 21-d-old tomato plants. Per plant, three leaflets were infested with mites: the terminal leaflet (at least 4 cm in length) of the youngest leaf possible; the second leaflet (i.e. non-terminal) of the second youngest leaf; and the second (i.e. non-terminal) leaflet of the third youngest leaf. A lanolin (Sigma-Aldrich Chemie B.V., Zwijndrecht, the Netherlands) barrier was made around the petiolule to prevent the mites from escaping. Uninfested control leaflets got the same lanolin barrier.

**Methods S2** Protocol for the extraction and quantification of phytohormones from tomato leaves.

Extraction and analysis of phytohormones from tomato (*Solanum lycopersicum* cv Castlemart) was performed using the procedure of Wu *et al*. (2007) with some minor modifications. In short, *c*. 200 mg of frozen leaf material was homogenized (Precellys 24, Bertin Technologies, Aix-en-Provence, France) in 1 ml of ethyl acetate which had been spiked with D_6_-SA and D_5_-JA (C/D/N Isotopes Inc, Canada) as internal standards with a final concentration of 100 ng ml^-1^. Tubes were centrifuged at 13000 rpm for 10 min at 4°C and the supernatant (the ethyl acetate phase) was transferred to new tubes. The pellet was re-extracted with 0.5 ml of ethyl acetate without internal standards and centrifuged for 10 min at 4°C at 13000 rpm. Both supernatants were combined and then evaporated to dryness on a vacuum concentrator (CentriVap Centrifugal Concentrator, Labconco, Kansas City, MO, USA) at 30^o^C. The residue was re-suspended in 0.5 ml of 70% methanol (v/v), centrifuged, and the supernatants were transferred to glass tubes and then analyzed by means of LC-MS/MS. A serial dilution of pure standards of OPDA, JA, JA-Ile and SA was run separately. Measurements were conducted on a liquid chromatography tandem mass spectrometry system (Varian 320-MS LC/MS, Agilent Technologies, Amstelveen, the Netherlands). We injected 20 µl of each sample onto a Pursuit XRs 5 column (C18; 50 × 2.0 mm, Agilent Technologies, Amstelveen, the Netherlands). The mobile phase comprised of solvent A (0.05% formic acid in water; Sigma-Aldrich, Zwijndrecht, the Netherlands) and solvent B (0.05% formic acid in methanol; Sigma-Aldrich). The program was set as follows: 95% solvent A for 1 min 30 s (flow rate 0.4 ml min^-1^), followed by 6 min in which solvent B increased till 98% (0.2 ml min^-1^) which continued for 2 min 30 s with the same flow rate, followed by 1 min 30 s with increased flow rate (0.4 ml min^-1^), subsequently returning to 95% solvent A for 1 min until the end of the run. A negative electrospray ionization mode was used for detection. The parent ions, daughter ions, and collision energies used in these analyses are listed in Table S1. For all oxylipins we used D_5_-JA to estimate the recovery rate and their *in planta* concentrations were subsequently quantified using the external standard series. For SA we used D_6_-SA to estimate the recovery rate and it was quantified using the external standard. Phytohormone amounts were expressed as ng per gram fresh mass leaf material.

**Note S1** Sampling and rearing of spider mites.

*Tetranychus evansi* Viçosa-1 was collected from tomato (*Solanum lycopersicum* cv Santa Clara) in a glasshouse on the campus of the Federal University of Viçosa, Brazil (GPS coordinates: 20 45.473 S 42 52.163 W) where it was maintained on detached leaves of the same tomato cultivar. It was previously shown to suppress JA- and SA-related defenses on these plants (Sarmento *et al*., 2011a). Ten heavily infested leaves from the Viçosa population were transferred to Amsterdam in 2009 and the mites were propagated on detached leaves of *S. lycopersicum* cv Castlemart ever since (see the Materials and Methods section). To explore whether suppression of defenses by *T. evansi* is a haplotype-specific trait we included a second haplotype, i.e. Algarrobo-1, which belongs to a different phylogenetic clade (Fig. S2a; Boubou *et al*., 2012). *Tetranychus evansi* Algarrobo-1 was collected in 2011 near Malaga, Spain (GPS coordinates: 36 45.487 N 4 02.407 W) from a single *S. nigrum* plant. These mites were transferred to Amsterdam in 2011 and propagated on detached leaves of *S. lycopersicum* cv Castlemart ever since. The base population from which isofemale strain *T. urticae* DeLier-1 was selected was sampled from deadnettle (*Lamium album*) near De Lier, the Netherlands in 2009 (GPS coordinates: 51 57.124 N 4 13.108 E). After the indicated selection procedure on *def-1*, WT and *35S::Prosystemin* tomato plants (see the Materials and Methods section), the *T. urticae* DeLier-1 strain was not reared on tomato anymore, but on detached leaves of the common bean (*Phaseolus vulgaris* cv Speedy) to prevent selection for mites resistant to tomato-induced defenses. *T. urticae* Santpoort-2, was an isofemale strain described previously in Kant *et al*. (2008) where it was referred to as ‘KMB’. It was collected from spindle tree (*Euonymus europaeus*) near Santpoort, the Netherlands (GPS coordinates: 52 26.503 N 4 36.315 E) in 2001 and has been propagated on detached *Phaseolus vulgaris* cv Speedy leaves ever since. This was done to prevent selection for mites that either suppress, or are resistant to, tomato-induced defenses.

**Note S2** The *Proteinase Inhibitor II* (*PI-II*) gene family in tomato.

We noticed that the transcript levels of the ‘classical’ marker *Proteinase Inhibitor IIf* (*PI-IIf*), also referred to as *Wound-Induced Proteinase Inhibitor II* (*WIPI-II,* Graham *et al*., 1985; Farmer *et al*., 1992; Li *et al*., 2002; Ament *et al*., 2004; Kant *et al*., 2004; Zhang *et al*., 2004; Kant *et al*., 2008) and which is highly induced by methyl jasmonate (MeJA)-treatment (Li *et al*., 2004) and by spider mites (Kant *et al*., 2004, 2008), did not always show a strong correlation with endogenous jasmonic acid (JA) levels (Fig. S5b,d,f,h), suggesting its expression may be regulated by additional signals. *WIPI-II* is one of six paralogous genes clustered adjacently in the genome (i.e. Solyc03g020030 until Solyc03g020080). We assessed the expression of the paralogs in correlation with endogenous JA-levels and selected Solyc03g020050 (Gadea *et al*., 1996), which we designated as *PI-IIc* since it is the third locus in the cluster, as a more reliable quantitative JA-marker (Fig. S5a,c,e,g).

**References**

**Ament K, Kant MR, Sabelis MW, Haring MA, Schuurink RC.** **2004.** Jasmonic acid is a key regulator of spider mite-induced volatile terpenoid and methyl salicylate emission in tomato. *Plant Physiology* **135:** 2025–2037.

**Boubou A, Migeon A, Roderick GK, Auger P, Cornuet JM, Magalhaes, S, Navajas, M.** **2012.** Test of colonisation scenarios reveals complex invasion history of the red tomato spider mite *Tetranychus evansi*. *PLoS One* **7:** e35601.

**Chen H, Wilkerson CG, Kuchar JA, Phinney BS, Howe GA. 2005.** Jasmonate-inducible plant enzymes degrade essential amino acids in the herbivore midgut. *Proc Natl Acad Sci, USA* **102:** 19237–19242.

**Farmer EE, Johnson RR, Ryan CA.** **1992.** Regulation of expression of proteinase-inhibitor genes by methyl jasmonate and jasmonic acid. *Plant Physiology* **98:** 995–100.

**Gadea J, Mayda ME, Conejero V, Vera P.** **1996.** Characterization of defense-related genes ectopically expressed in viroid-infected tomato plants. *Mol Plant-Microbe Int* **9:** 409–415.

**Graham JS, Pearce G, Merryweather J, Titani K, Ericsson LH, Ryan CA.** **1985.** Wound-induced proteinase inhibitors from tomato leaves. II. The cDNA-deduced primary structure of pre-inhibitor II. *J Biol Chem* **260:** 6561–6564.

**Itkin M, Rogachev I, Alkan N, Rosenberg T, Malitsky S, Masini L, Meir S, Iijima Y, Aoki K, de Vos R *et al*.** **2011.** GLYCOALKALOID METABOLISM1 is required for steroidal alkaloid glycosylation and prevention of phytotoxicity in tomato. *Plant Cell* **23:** 4507–4525.

**Kant MR, Ament K, Sabelis MW, Haring MA, Schuurink RC. 2004.** Differential timing of spider mite-induced direct and indirect defenses in tomato plants. *Plant Physiology* **135:** 483–495.

**Kant MR, Sabelis MW, Haring MA, Schuurink RC.** **2008.** Intraspecific variation in a generalist herbivore accounts for differential induction and impact of host plant defences. *Proc R Soc B* **275:** 443–452.

**Koo AJK, Gao X, Jones AD, Howe GA.** **2009.** A rapid wound signal activates the systemic synthesis of bioactive jasmonates in *Arabidopsis*. *Plant Journal* **59:** 974–986.

**Lers A, Sonego L, Green PJ, Burd S.** **2006.** Suppression of LX Ribonuclease in tomato results in a delay of leaf senescence and abscission. *Plant Physiology* **142:** 710–721.

**Li C, Williams MM, Loh YT, Lee GI, Howe GA.** **2002.** Resistance of cultivated tomato to cell content-feeding herbivores is regulated by the octadecanoid-signaling pathway. *Plant Physiology* **130:** 494–503.

**Li L, Zhao Y, McCaig BC, Wingerd BA, Wang J, Whalon ME, Pichersky E, Howe GA.** **2004.** The tomato homolog of CORONATINE-INSENSITIVE 1 is required for maternal control of seed maturation, jasmonate-signaled defense responses, and glandular trichome development. *Plant Cell* **16:** 126–143.

**Lin Q, Hamilton WDO, Merryweather A.** **1996.** Cloning and initial characterization of 14 myb-related cDNAs from tomato (*Lycopersicon esculentum* cv Ailsa Craig). *Plant Mol Biol* **30:** 1009–1020.

**Lisón P, Rodrigo I, Conejero V. 2006.** A novel function for the cathepsin D inhibitor in tomato. *Plant Physiology* **142:** 1329–1339.

**Newman SM, Eannetta NT, Yu HF, Prince JP, Devicente MC, Tanksley SD, Steffens JC.** **1993.** Organization of the tomato polyphenol oxidase gene family. *Plant Mol Biol* **21:** 1035–1051.

**Sarmento RA, Lemos F, Bleeker PM, Schuurink RC, Pallini A, Oliveira MGA, Lima E, Kant M, Sabelis MW, Janssen A.** **2011a**. A herbivore that manipulates plant defence. *Ecology Letters* **14:** 229–236.

**Sokal RR, Rohlf FJ.** **2012.** Linear Regression. In: RR Sokal, FJ Rohlf, eds. *Biometry*, *4th edn*. New York, NY, YSA: W. H. Freeman and Company, 471–549.

**Tamura K, Peterson D, Peterson N, Stecher G, Nei M, Kumar S.** **2011.** MEGA5: molecular evolutionary genetic analysis using maximum likelihood, evolutionary distance and maximum parsimony methods. *Mol Biol Evol* **28:** 2731–2739.

**Tomato Genome Consortium.** **2012.** The tomato genome sequence provides insights into fleshy fruit evolution. *Nature* **485:** 635–641.

**Van Kan JAL, Joosten MHAJ, Wagemakers CAM, Van den Berg-Velthuis GCM, de Wit PJGM.** **1992.** Differential accumulation of messenger-RNAs encoding extracellular and intracellular PR proteins in tomato induced by virulent and avirulent races of *Cladosporium-fulvum*. *Plant Mol Biol* **20:** 513–527.

**Wu J, Hettenhausen C, Meldau S, Baldwin IT.** **2007.** Herbivory rapidly activates MAPK signalling in attacked and unattacked leaf regions but not between leaves of *Nicotiana attenuata*. *Plant Cell* **19:** 1096–1122.

**Zhang HY, Xie XZ, Xu YZ, Wu NH. 2004.** Isolation and functional assessment of a tomato proteinase inhibitor II gene. *Plant Physiol Biochem* **42:** 437–444.
